# Proteins as templates for complex synthetic metalloclusters: towards biologically programmed heterogeneous catalysis

**DOI:** 10.1098/rspa.2016.0078

**Published:** 2016-05

**Authors:** Charlie Fehl, Benjamin G. Davis

**Affiliations:** Department of Chemistry, University of Oxford, Oxford OX1 3TA, UK

**Keywords:** artificial metalloproteins, heterofunctional metalloclusters, hydrogenase engineering, iron–sulfur proteins, heterometallic proteins, de novo design

## Abstract

Despite nature’s prevalent use of metals as prosthetics to adapt or enhance the behaviour of proteins, our ability to programme such architectural organization remains underdeveloped. Multi-metal clusters buried in proteins underpin the most remarkable chemical transformations in nature, but we are not yet in a position to fully mimic or exploit such systems. With the advent of copious, relevant structural information, judicious mechanistic studies and the use of accessible computational methods in protein design coupled with new synthetic methods for building biomacromolecules, we can envisage a ‘new dawn’ that will allow us to build de novo metalloenzymes that move beyond mono-metal centres. In particular, we highlight the need for systems that approach the multi-centred clusters that have evolved to couple electron shuttling with catalysis. Such hybrids may be viewed as exciting mid-points between homogeneous and heterogeneous catalysts which also exploit the primary benefits of biocatalysis.

## 1. Introduction: natural metalloprotein inspirations for synthetic templates

The ability of transition metals to catalyse chemical transformations is used in almost all areas of industry and energy [1]. Equally important is the prevalence of metals in enzymes, the biological catalysts that underpin life. Metals are present in almost half of the proteins found in nature [2]. The processes that metalloproteins carry out, from the complex organic chemistry of building natural products to the seemingly simple reactions of water oxidation, carbon dioxide reduction and nitrogen fixation have wholly reshaped the planet and its atmosphere.

An emerging strategy to direct the power of metalloproteins into new applications has been the design of protein scaffolds to use a variety of metals [3], including non-natural metals [4]. Some of these have been successful, and the outcomes of these studies have contributed not only improved biocatalysts but also a wealth of understanding in protein design, folding and optimization [5]. However, the vast majority of these efforts have focused on adding single metals to a protein scaffold [3]. In many natural transformations complex metalloenzymes, such as the photosynthetic complexes photosystem I and II (PSI and PSII) [6], the nitrogen-fixing nitrogenases [7] and the hydrogen-producing di-iron hydrogenases [8], rely on multi-metal motifs: catalytic sites as well as metal-based electron- or proton-delivery systems. Thus, heterometallic and multi-metallic proteins, which bind different types of metals or clusters of metals, represent an overarching unmet challenge in artificial metalloprotein design.

Truly effective mimics of such ‘complex metalloproteins’ (the ‘catch-all’ term that we will use herein for metalloproteins with two or more varied metal sites or clusters) have not yet been developed. As such, this remains a key goal in synthetic enzymology. The benefits of incorporating hetero/multi-metallic sites could be profound: de novo variants of important biological processes could be developed [9] and new reactivity from motifs discovered [10]. In this review, we present an overview of recent research building towards those goals.

**Figure 1.**
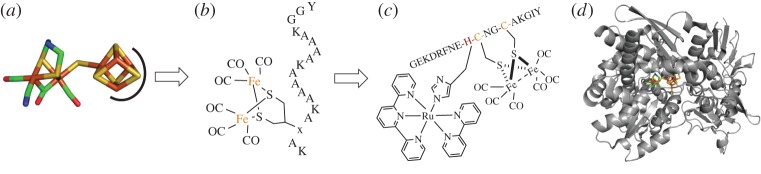
Approaches towards mimics of metalloproteins. (*a*) Organometallic di-iron system. Extracted from PDB ID:4XDC [11]. Iron is shown in orange, sulfur in yellow, carbon in green, nitrogen in blue and oxygen in red. (*b*) Peptide model in a 19 amino acid residue helical system [12]. Peptide residues are shown as their one-letter amino acid codes. (*c*) Bimetallic peptide system increases the complexity [13]. (*d*) Biological hydrogenases have more than five orders of magnitude higher activity than synthetic systems. Shown here is *Clostridium pasteurianum* [FeFe] hydrogenase (PDB ID: 4XDC) [11].

In particular, we highlight a progression for protein design around three types of metalloclusters that comprise major synthetic foci: iron–sulfur [FeS], di-iron [FeFe] and nickel–iron [NiFe] clusters. Following the structural determination of these clusters in biological active sites, simple organometallic mimics of the metalloclusters themselves (figure 1*a*) have led to larger peptide systems that more accurately mimic metalloprotein activity. In a select few examples, complete heterometallic complexes have been built (figure 1*c*) in an attempt to mimic full metalloprotein systems (figure 1*d*). We hope that this review inspires continued work towards the development of multi-metal sites in new peptide- and protein-based catalysts that move beyond Ni and Fe toward metals that are rarely or never found in nature [4].

## 2. Models of protein metalloclusters: from organometallic systems to peptide frameworks

The inherent complexity of multi-centred metalloproteins presents a major challenge toward understanding the functions of their individual components. Rather than attempting to study full systems, the standard approach, thus far, has been the construction of simplified models to understand the various pieces of multi-faceted metalloclusters [14]. Although models are often unable to fully explain a system, the prospect of extracting complete or partial metal centres from protein architectures for synthetic use has driven extensive efforts to build bio-inspired mimics of these active sites [15]. Over 300 mimetics of the iron–iron cluster ([FeFe]) of the di-iron hydrogenases alone have been synthesized, with or without pendant electron acceptor systems designed to approximate the natural activities of these enzymes [15]. Several other metalloclusters have been targeted for similar synthetic studies [16].

Despite enormous efforts to replicate the structure and function of complex metalloclusters outside of protein active sites, significant limitations occur in the activity, selectivity and stability of small organometallic ligand systems compared with their protein scaffolds. One approach towards improving these parameters has been the use of short (*ca* 6–50 amino acid residues) peptide backbones [17,18]. Peptides based on natural metalloprotein motifs can adopt suitable geometries to accommodate metal centres, and by their amino acid-based nature clearly use the same building blocks as natural protein active sites. Furthermore, peptides shorter than 50 amino acid residues can often be prepared on an automated peptide synthesizer, circumventing the need for biological production.

In this section, we highlight efforts to model three metalloclusters: iron–sulfur ([FeS]) clusters, di-iron ([FeFe]) clusters and nickel–iron ([NiFe]) clusters. For all three cases, natural binding motifs have inspired the design of compact organometallic frameworks, which have in turn informed the construction of peptide-based systems. Such studies continue to inform the design of more complex peptide and even the design of artificial proteins with an aim towards approaching the activities of natural metalloprotein systems.

### (a) Iron–sulfur clusters

Owing to the catalytic versatility of iron–sulfur clusters in biology, various types of [FeS] clusters have long been targeted for synthesis and mimicry [19]. Broadly speaking, these systems serve as conduits for electrons, both within an individual protein core as well as across protein–protein interfaces. [FeS] clusters are most identifiably associated with dedicated electron-transferring proteins such as the ferredoxins and adrenodoxins, which shuttle electrons within cellular compartments [20]. [FeS] clusters are also crucial cofactors of many redox-active protein centres, where they can modulate redox potentials to accommodate their substrates. Notably, several substituents of the photosystem complexes (PSI and PSII) contain one or more [FeS] clusters, which act as electron channels during the conversion of visible light to chemical energy [16].

**Figure 2.**
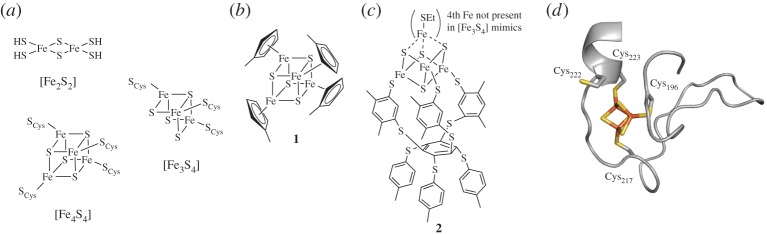
Iron–sulfur clusters and selected mimics. (*a*) Three common [FeS] clusters in biology. (*b*) An early structural mimic of [Fe_4_S_4_] clusters [21]. (*c*) Advanced mimics of [Fe_3_S_4_] and [Fe_4_S_4_] clusters in a cavitand of defined geometry. (*d*) Peptide ‘maquettes’ based on a 33-residue [FeS]-binding motif of DmsB. The structure of a related motif from *Escherichia coli* nitrate reductase is shown with an [Fe_3_S_4_] cluster (PDB ID: 1Q16, residues 193–226 of chain B [22]). These analogues were used to probe the function of each labelled cysteine as well as nearby residues in the context of cluster stability [18]. The peptide backbone is shown in grey (X-ray structure of an [FeS] binding protein, PDB ID: 1Q16), iron atoms as orange and sulfur atoms as yellow.

Proteins can harbour different types of [FeS] clusters by constraining the geometries of [FeS] binding domains. The most stable clusters are [Fe_2_S_2_] and [Fe_4_S_4_], though [Fe_3_S_4_] clusters are also employed in biology (figure 2*a*). Clusters are typically bound through the thiol of cysteine residues (S_Cys_), though occasionally other heteroatom-containing amino acids can act as ligands. The composition and protein environment of [FeS] clusters finely tunes the redox potentials of individual clusters, allowing directional electron transport cascades [20].

Structural, as opposed to functional, mimics of simple [Fe_4_S_4_] complexes have been readily formed by refluxing cyclopentadienyl (Cp) iron species such as Fe(methyl-Cp)_2_(CO)_2_ with an excess of sulfur to form **1** (figure 2*b*) [21]. However, a more rigid system that also allowed the study of [Fe_3_S_4_] versus [Fe_4_S_4_] states was presented by Holm and co-workers [23]; this used the cavitand formed by the hexathiobenzene of compound **2** to bind three corners of the cubane [Fe_X_S_4_] structure (figure 2*c*). Clusters based on **2** quite closely matched the spectroscopic profile of the respective natural [Fe_3_S_4_] and [Fe_4_S_4_] centres, with a sulfur ligand of the fourth iron (if present) comprising a low molecular weight thiol.

More advanced mimics to study the structure of [Fe_3_S_4_] and [Fe_4_S_4_] have since been built within peptide frameworks. Lubitz *et al.* have presented a series of mimics of the 33-residue cluster-binding peptide (CBP) domain of a bacterial dimethyl sulfoxide reductase subunit B (DmsB). These mimics were used to probe the structural and functional roles of the various residues in this small peptide domain (figure 2*d*) [18]. Certain sequences which kinetically favoured [Fe_3_S_4_] over the more thermodynamically stable [Fe_4_S_4_] clusters were observed, though [Fe_4_S_4_] formation ultimately dominated, suggesting a potentially stepwise formation of [Fe_4_S_4_] via an initial [Fe_3_S_4_] complex in this peptide.

Previously, the same group had reported a series of de novo designed peptides based on two distinct [FeS]-binding components of PSI. These were shorter in sequence at 16 residues each, and closely matched the natural reduction potential of –0.465 V with −0.440 V for the F_A_ motif of PSI and the designed peptide, respectively [24]. Despite their minimized organization, these efforts highlight the improving abilities of de novo design to replicate or create novel metal-binding sites in peptides and proteins [2].

Thus, synthetic [FeS] systems have been characterized for their structures and redox potentials, and continue to inform the design of small protein domains to harbour [FeS] clusters with designed properties. These studies serve as an essential step towards building complex and truly effective electron-shuttling systems in peptide frameworks (*vide infra*). However, as [FeS] clusters do not display inherent catalytic activity, these reports have so far mainly served as proofs-of-concept that await coupling to a chemically reactive site.

### (b) Di-iron clusters

One group of redox enzymes that typically use [FeS] clusters to obtain electrons for catalysis is the di-iron ([FeFe]) hydrogenase class. These enzymes have multiple activities, but mainly catalyse proton reduction to hydrogen (H_2_ production) as well as the reverse process (H_2_ oxidation). These activities allow microorganisms to use H_2_ as a sink or source of electrons, respectively [15]. In some systems, hydrogen oxidation can be coupled to further reactions such as the reduction of carbon dioxide to methane [25].

These enzymes often have remarkably high activities, especially given the fact that they operate under ambient aqueous conditions without any applied electric potentials. Although difficult to study due to fast rates, a bacterial [FeFe] hydrogenase from *Clostridium acetobutylicum* was immobilized onto an electrode to approximate activity using single molecule studies. Armstrong *et al.* thus observed a turnover frequency (TOF) of approximately 21 000 molecules of H_2_ s^−1^ at pH 7.0 [26].

**Figure 3.**
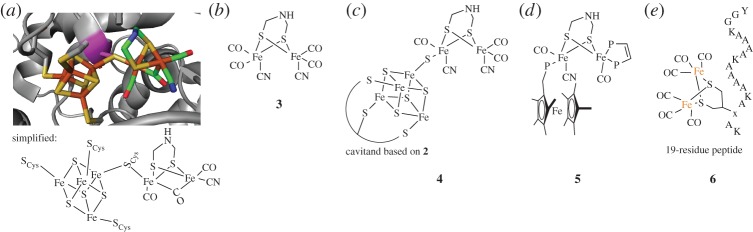
Di-iron clusters and selected mimics. (*a*) The natural [FeFe] cluster of *Clostridium pasteurianum* [FeFe] hydrogenase (PDB ID: 4XDC) [11]. The attached [Fe_4_S_4_] group in this hydrophobic pocket make up the so-called H-cluster. Protein backbone shown in grey, the bridging cysteine residue in magenta. Sulfur atoms shown in yellow, iron in orange, oxygen in red, nitrogen in blue and carbon in green. (*b*) Prototypic ‘simple’ mimic of the [FeFe] cluster, with natural CO and CN ligands. (*c*) An H-cluster mimic incorporating the [Fe_4_S_4_] cavitand of compound **2** (full ligand system abbreviated). (*d*) The only catalytic [FeFe] mimic to function without an applied electrical overpotential. This uses a ferrocene moiety as the electron acceptor and two abiological phosphine ligands. (*e*) A representation of a peptide-linked [FeFe] cluster used for the photochemical production of H_2_. The helical peptide sequence is shown in one-letter amino acid code, with the unnatural dithiol amino acid shown as only the sidechain (labelled ‘x’ in the sequence).

The structures of several [FeFe] clusters in proteins have been determined using X-ray crystallography, with an example shown in figure 3*a* [11]. The [Fe_4_S_4_] cluster and the [FeFe] cluster, bridged by a single cysteine thiol, together make up the so-called H-cluster of these enzymes.

As mentioned above, hundreds of structural and functional mimics of [FeFe] systems have been synthesized. An early generation of these mimics was created that used the biological ligands carbon monoxide (CO) and cyanide (CN) (figure 3*b*). Variants of these complexes were able to reduce protons, but required electrical potentials of larger than −1.01 V to be applied [8]. This is a far cry from natural [FeFe] systems, which do not require any applied potential in order to function. Reducing the magnitude of these overpotentials, defined as the extra energy above the standard H^+^/H_2_ redox couple required for reaction, is a key target in the design of analogues.

To improve the activities of these mimics, redox-active centres have been appended to the [FeFe] sites in an attempt to lower the electric potential required for activation. One strategy, used by Tard *et al.* [27], attached the [Fe_4_S_4_] cavitand **2** to mimic the natural [FeS]–[FeFe] H-cluster complex (compound **4**, figure 3*c*). However, **4** still required an overpotential of −0.96 V for activity, providing only a marginal improvement of 0.05 V over prior mimics such as **3**.

The first [FeFe] mimic that did not require an applied overpotential for catalytic H_2_ oxidation was compound **5** (figure 3*d*) [28]. The decamethylferrocene electron acceptor, though unnatural, provided the first reported catalytic turnover for this reaction in the presence of excess chemical oxidant FcBAr^F^_4_. However, the TOF of **5** is 10^−4^ molecules H_2_ s^−1^, which pales in comparison to natural turnovers upwards of 20 000 molecules H_2_ s^−1^.

Similar to the models of [FeS] clusters, key progress in improving the structure and in this case TOF of [FeFe] metallocluster mimics has been observed when anchored within peptide frameworks. One potential reason for an improvement in activity is the conserved hydrophobic pocket in which the [FeFe] clusters reside, which shields them from an aqueous environment [29]. Site-directed mutagenesis has also identified contributions of key individual amino acids to the electronic structure and high TOF of these metalloclusters, suggesting that certain geometries and ligands lead to higher activity [29]. A prominent example of the anchoring of an [FeFe] cluster to a short peptide is shown in compound **6** (figure 3*e*) [12]. This 19-residue short helical peptide with a dithiol bridging motif was catalytically active for the reverse process, hydrogen production. Compound **6** was able to provide a TOF of 0.61 molecules H_2_ s^−1^, though this reaction required a photosensitizer and ascorbic acid as the electron donor. Similar strategies reviewed elsewhere [15] involve full or partial cytochrome *c* protein domains, but cytochrome *c*-containing catalysts displayed reduced activity compared with **6**.

The ability to structurally mimic [FeFe] clusters has thus proceeded further than the ability to create active *functional* mimics of these metalloclusters for hydrogen oxidation and production. However, the continued design of these catalysts has provided useful insight into the required properties of active synthetic catalysts. Moreover, clear limitations of these systems outside of a protein framework have been noted. Namely, it has proved difficult to sequester these metalloclusters in a hydrophobic pocket or fine-tune their electronic structures using organometallic systems alone. The use of peptides is therefore a promising strategy for better mimicking [FeFe] hydrogenases that not only moves towards biomimicry, but is more pragmatic—small, readily synthesized helical domains appear to be sufficient. Short ‘protein-inspired’ domains have thus proved sufficient for incorporating active [FeFe] clusters into peptidic systems in a modular fashion, though their activities must still be improved.

### (c) Nickel–iron clusters

A related group of metalloproteins to the [FeFe] hydrogenases are the [NiFe] hydrogenases. These enzymes also catalyse the oxidation of hydrogen, providing TOFs of 1500–9000 molecules H_2_ s^−1^ without the need for applied electric overpotentials [30]. The [NiFe] cluster is a heterometallic system with distinct geometry from the [FeFe] metalloclusters, offering a complementary solution to the challenge of hydrogen activation and production [31]. These clusters have proved more challenging to functionally mimic using simple organometallic systems, which again suggests the need for a complex scaffold to obtain high activity.

**Figure 4.**
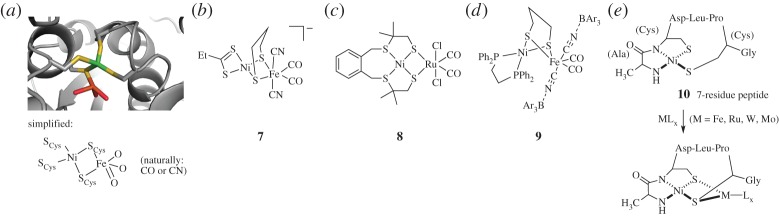
Nickel–iron clusters and selected mimics. (*a*) The [NiFe] cluster of *Desulfovibrio gigas*[NiFe] hydrogenase (PDB ID: 1FRV). Protein backbone shown in grey. Sulfur atoms shown in yellow, iron in orange, nickel in green and oxygen in red. (*b*) The closest structural mimic, based on Ni–S angles, Ni–Fe distance and Fe ligand system [33]. (*c*) An early functional mimic for H_2_ oxidation [31]. (*d*) The first Ni/Fe-based functional mimic, requiring abiological ligand systems. (*e*) A short peptide designed to bind heterometallic species.

Structural mimics were developed shortly after the first crystal structures of [NiFe] enzymes were reported (reviewed elsewhere [31,32]). Figure 4*a* shows a representation of an [NiFe] cluster from the core of the *Desulfovibrio gigas* [NiFe]-hydrogenase [33]. In contrast with the [FeS] and [FeFe] models, however, direct structural mimics of [NiFe] clusters fail to generate functional activity. In particular, it has proved difficult to approximate three biological properties at once: the short Ni–Fe distance of 2.6–2.9 Å, the distorted, non-square planar geometry of the active Ni species, and the biologically relevant CO/CN ligands at the iron site. Compound **7**, synthesized by the Tatsumi group, approaches all three parameters (figure 4*b*) [34]. However, no Ni/Fe mimic has successfully recapitulated activity for either hydrogen oxidation or production using biological ligands at Ni and Fe.

One strategy for functional hydrogen evolution catalysts as [NiFe] mimics has been the replacement of Fe with the noble metal ruthenium (figure 4*c*) [31]. Compound **8** was indeed able to produce H_2_ with a TOF of 10 molecules H_2_ s^−1^, albeit at a large overpotential of −1.2 V [35]. Interestingly, Ni/Fe constructs using the same ligand system of **8** failed to show any activity [31].

Although biological structural/functional mimics of [NiFe] clusters have not yet been prepared, Manor & Rauchfuss [36] have reported a biomimetic Ni–Fe system that approaches both goals, albeit with abiological ligands such as phosphines (figure 4*d*). Compound **9** can carry out both H_2_ oxidation and H_2_ production, similar to the natural system. The latter process, however, only occurs on a stoichiometric level with regard to **9**. Despite shortcomings, this is a promising step toward active biomimetic [NiFe] clusters.

A strategy based on incorporating [NiFe] clusters into peptide systems was recently reported by the Jones laboratory [17]. In this work, a short heptapeptide ‘nickel-binding hook’ was mined from the enzyme nickel superoxide dismutase. This 7-residue ‘NiSODA’ peptide was evaluated for peptide-Ni-[second metal] heterometallic complex formation, shown in compound **11** (figure 4*e*) [17]. The second metals used included Mo, W, Ru and Fe. Although these complexes were not characterized for functional activity, the specificity of the nickel-binding motif in the NiSODA peptide appears of promising use in the creation of heterobimetallic species.

These examples illustrate the ability to replicate the natural activities of three types of metalloclusters in simplified model systems. A key emergent theme is the ability to reconstruct full systems within natural or designed peptide sequences, which for [FeS] and [FeFe] clusters led to improvements in approximating useful redox ability or activity. However, the pre-eminent benefit of using short, designed peptide domains to harbour metalloclusters is arguably their modular construction. As in nature, these domains can be potentially linked together, providing protein scaffolds with combined functionalities. Such a strategy may be a starting point for overcoming the limitations in activity observed with simpler models of metalloclusters to date.

## 3. Progress towards the installation of multiple metallic centres in peptide scaffolds for redox cascades

The fine control of electron delivery into a metallocluster is likely a major factor in the efficiency of natural metalloenzymes, and one which has been investigated in theoretical models of [FeFe] systems containing [FeS] clusters [37]. These types of ‘molecular-wire’ systems are exquisitely complex, but attempting their re-creation presents an opportunity to both improve designed metalloproteins and to better study how native systems function. Indeed, this opportunity is presently being addressed in several proof-of-concept systems. To date, many short peptides and even full proteins have been designed as artificial metalloenzymes—this work is excellently reviewed elsewhere [3]. However, these systems have been, for the most part, designed around a single metal site, or two symmetrical ones in the case of the designed di-iron *due* *ferri* proteins [38]. More rare are studies that combine electron-transferring capabilities with catalytic functionality, which is the focus of this section.

**Figure 5.**
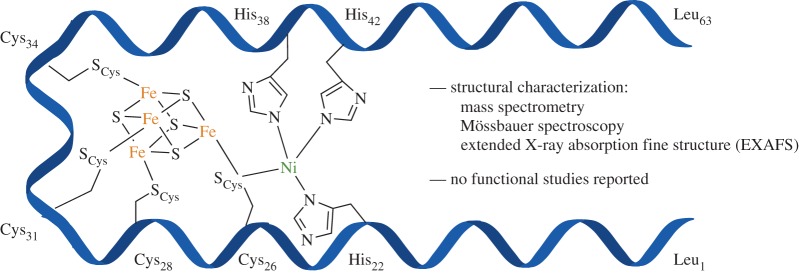
A cartoon representation of the helix–loop–helix peptide designed to bind an [Fe_4_S_4_] cluster and an Ni ion. A more accurate peptide structural model was constructed with Quanta, included in the original report [39].

In an ambitious construct conceptually based on the [FeS]-Ni motif, or the so-called A-cluster, of carbon monoxide dehydrogenase (CODH), Laplaza & Holm [39] designed a helix–loop–helix peptide motif to bind an [Fe_4_S_4_] cluster and a Ni ion. As depicted in figure 5, this 63-residue peptide rigidly holds an [Fe_4_S_4_] and contains three nearby histidines that position a Ni ion in the correct proximity to form a shared thiolate bridge. Thus, this small peptide unit resembled the A-cluster of CODH. This construct was examined with Mössbauer spectroscopy and extended X-ray absorption fine structure (EXAFS) to confirm metal identity and stoichiometry. Additionally, circular dichroism spectroscopy indicated subtle secondary structural changes upon both [Fe_4_S_4_] and Ni additions. However, this construct was not evaluated for any electron transfer activity, perhaps because it displayed marked instability upon treatment with the reducing agent dithionite.

**Figure 6.**
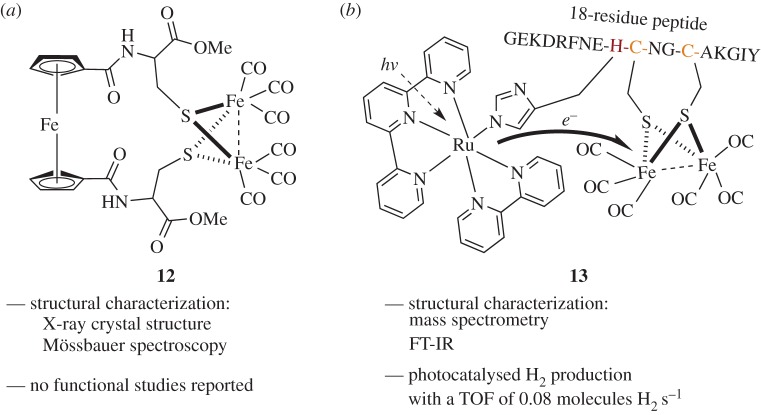
Peptide-based H-cluster mimics of [FeFe] hydrogenases. (*a*) A simple ferrocene-cysteine mimic used an abiological electron acceptor [40]. (*b*) A cartoon of an 18 residue peptide containing an [FeFe] cluster and an [Ru] photosensitizer complex showed the ability to harvest and transfer electrons, but again used an abiological electron donor [13].

A model H-cluster of the [FeFe] enzymes was constructed by de Hatten *et al*. Here, peptide-substituted cyclopentadiene units were used to construct a ferrocene-type molecule bound by cysteines to an [FeFe] cluster (compound **12**, figure 6*a*) [40]. While **12** does not use a biomimetic system due to the ferrocene electron acceptor, the peptide-based [FeFe] cluster is a step toward the goal of modelling the [FeFe]–S–[FeS] active site of the [FeFe] hydrogenases. The structure of **12** was determined by crystallography, but **12** was not investigated for catalytic activity.

A functional mimic of the H-cluster of an [FeFe] hydrogenase in a protein-based environment was constructed by Hayashi *et al*. An 18-residue peptide containing the CxxCH motif of cytochrome *c*_556_ was chosen for its ability to bind an [FeFe] cluster. Additionally, this peptide contains a histidine residue, which can bind a Ru(bpy)(tpy) complex to act as a photosensitizer for electron production [13]. The full construct is represented in figure 6*b* as compound **13**. Thus, this work is similar to the work of Jones and co-workers toward compound **6**, but incorporates the [FeFe] cluster and an [Ru] photosensitizer on the same molecule as opposed to using the latter in solution. Interestingly, the TOF of **13** was significantly lower than **6**, at 0.08 molecules s^−1^ versus 0.6 molecules s^−1^, respectively. A control peptide lacking the Ru-coordinating histidine residue failed to perform H_2_ evolution, even when exogenous soluble ruthenium was added as Ru(bpy)_3_. The reason for this inactivation is not clear, but suggests that in the peptide scaffold of **13**, the distance between the electron-donating moiety and the [FeFe] cluster may be critical.

**Figure 7.**
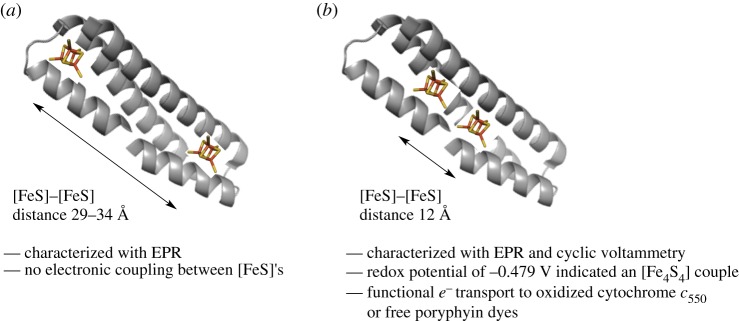
Peptide mimics of coupled [FeS] clusters. (*a*) Representation of the first generation three-helix bundle peptide DSD redesigned to bind two [Fe_4_S_4_] clusters. (*b*) Representation of an improved DSD mimic able to transfer electrons to proteins as well as a small molecule dye. Peptide backbone in grey, Fe atoms in orange and sulfur in yellow. Molecular models of each are presented in [41,42].

Finally, more complex mimics of [FeS] proteins have been reconstituted in larger peptides to approximate the ‘molecular wires’ used by many metalloprotein systems. In an approach that used the pseudo-twofold symmetry of a designed protein scaffold, Roy *et al.* [41] were able to insert two [Fe_4_S_4_] clusters into a three-helix bundle (figure 7*a*). This protein, DSD-bis[4Fe–4S], was modelled to suggest a 29–34 Ådistance between clusters. Electron paramagnetic resonance (EPR) studies were carried out to characterize the electron-transferring ability of these clusters. Pulsed electron–electron double resonance (ELDOR) experiments demonstrated that there was a weak interaction between the clusters. Although not necessarily conducive to efficient electron tunnelling, this laid useful groundwork for more advanced electron-relay systems.

Following this work, the DSD-based [FeS] system was improved by shortening the inter-cluster distance to the more biologically relevant 12 Å[42]. The resulting peptide, designed to mimic ferredoxin, was termed DSD-Fdm (figure 7*b*). The redox potential of the two [Fe_4_S_4_] clusters was found to be −0.479 V, which falls within the lower range of natural ferredoxins [20]. Furthermore, the authors showed that reduced DSD-Fdm could transfer an electron to oxidized cytochrome *c*_550_, a natural ferredoxin substrate, in a stoichiometric fashion. Thus, designed peptide systems have the ability to transfer electrons within and between proteins. This study demonstrates that redox components can be built in a modular fashion, greatly increasing the potential capabilities of new, designed protein systems.

Significant steps have been taken toward combining electron-relaying centres with catalytic metal sites. Although constructs built to mimic metalloenzymes to-date have yet to demonstrate significant catalytic activity, each system has provided valuable insights into the design and structure of metalloclusters. Additionally, the design of electron-transferring peptides has exciting implications for improving these systems by fine-tuning their redox potentials. Together, these findings serve as excellent building blocks toward a clearer understanding of multi-centred metalloproteins.

## 4. Summary: how to move from simple mimics of metalloclusters to heterometallic protein design?

The abilities of natural metalloproteins to catalyse some of the most important processes on the planet serve as an excellent inspiration for functional mimicry. Several mimics of the metalloclusters that these proteins use to carry out these functions have been synthesized to better understand their mechanisms and capabilities. However, the activities of these metalloclusters essentially fail when removed from their natural protein environments.

The move from small organometallic species toward larger peptide-based mimics is a promising direction toward advancing these studies. Peptidic mimics are better able to recapitulate some of the redox properties of the [FeS] clusters and, as enzyme mimics, show higher catalytic activities than many organometallic complexes described to-date. This may represent an intermediate step between the smallest possible metallocluster mimics and large protein systems intransigent to rational design. At the very least it highlights the beneficial properties (perhaps, simply of increased organization, stability and solubility) of the folds that are accessible in peptides. However, it must be acknowledged that the reduced complexity of these peptide mimics only allows hints of natural metalloprotein activities.

How might improvement best be achieved? Fortunately, a growing trend toward combining multiple metallocluster-based functionalities into single molecules is occurring. Peptide-based systems again serve as the pre-eminent template, as they can be iteratively linked to connect multiple domains in a modular fashion. Such ‘bottom-up’ models of advanced heterobimetalloclusters, such as the [FeS]/[Ni]-containing A-clusters of the CODH, represent an excellent proof-of-concept in this realm. Nonetheless, the design goals for these projects reflect an enormous increase in complexity and ambition in the scope of artificial metalloprotein creation when compared with the simple mimics of single metalloclusters that dominated the earlier literature in this field. At some point, issues of permutation complexity might create blind avenues for such modular approaches to approach the truly useful tertiary structures that we may need. To this end, it is notable that a largely untried approach has been one of ‘top down’. Thus, while some inspiring examples of the redesign of natural metalloprotein as ‘boxes’ for metals exist [43], their exploitation for catalytic heterometalloclusters is not yet demonstrated. Coupled with some success recently in computational metalloprotein design [44], some promising new ways forward can be envisaged.

Thus, we are entering an exciting era for the construction of artificial metalloproteins of increasingly broad relevance to homogeneous and even heterogeneous ‘chemical’ catalysis. These systems continue to push the boundaries of our abilities to mimic complex metalloproteins and to erode the perception that somehow biocatalysis is ‘not chemical’ or is ‘in some way cheating’. There is enormous room for improvement by replicating or mimicking natural systems that have benefited from evolutionary design, but nonetheless the recent progress of this field in embracing more complex peptide-based systems indicates a steady aim in what we consider to be the ‘right’ direction.
